# Supplementary material for: Biomarkers Related to Synaptic Dysfunction to Discriminate Alzheimer’s Disease from Other Neurological Disorders
Source: Int J Mol Sci. 2022 Sep 16;23(18):10831. doi: 10.3390/ijms231810831 (PMC9501545; doi:10.3390/ijms231810831)
Supplement: Supplementary file 1 [file ijms-23-10831-s001.zip › ijms-1859291-supplementary.pdf]

Supplementary materials

Table S1- List of Neurodegenerative disorders of n-AD patients**.**

**Disorders**	**no.**
Cerebral Degeneration due to Iron accumulation	1
Cortico-Basal Degeneration (CBD)	10
Frontotemporal Dementia (FTD)	20
Lewy’s bodies Dementia (LBD)	10
Multisystemic Atrophy (MSA)	1
Parkinson’s disease (PD)	7
Pick’s Dementia	1

Table S2- List of neurological disorders of n-ND patients.

Disorders	no.
Neuralgia	3
Dystrophy	2
Psychic disorders	11
Hemorrhages/Tumor	5
Encephalopathies	7
Epilepsy	4
Normal Pressure Hydrocephalous	15
Ischemia	3
Leukoencephalopathies	3
Myelitis	1
Myelopathy	10
Neuropathy/Polyneuropathy	29
Forestier's syndrome	1
Multiple Sclerosis	1
Fibromyalgia	1
Myopathy	2

Table S-3. CSF levels of AD-related biomarkers (i.e., Aβ 42, pTau, tTau, and Aβ 42/40) in AD and n-AD patients.

**Variables**	**AD (n=69)**	**non-AD (n=50)**	**Size effect (η^2)**	***p***
Aβ 42 (*pg/ml*)	600 (443 – 778)	763 (558.5 – 1150.5)	0.067	0.017
Aβ 42/40 ratio	0.051 (0.040 – 0.079)	0.098 (0.072 – 0.102)	0.233	**<0.001**
pTau (*pg/ml*)	77.2 (43.5 – 97.9)	30.6 (20.3 – 39.5)	0.305	**<0.001**
tTau (*pg/ml*)	562 (402 – 798)	269 (192 – 375)	0.273	**<0.001**

Data are expressed as median with interquartile ranges (IQR) and are analyzed by Mann-Whitney U-test, also indicating the size effect (η2). P-values are corrected for multiplicity by the Holm-Sidak method and are indicated with bold font.

Table S-4. Coefficients of linear regression models testing association of Ng, α-Syn, Aβ42/Ng or Aβ42/ α-Syn with clinical features and other AD-related biomarkers in AD group.

Variables	Unstandardized Coefficients		Standardized Coefficients	T	p
	B	Standard error	beta		
Ng (cost.)					
MMSE <24	247.759	71.299	0.563	3.475	0.034
Aβ 42/40 <0.062	223.797	61.016	0.450	3.668	0.001
pTau>61pg/ml	216.011	50.475	0.530	4.280	<0.001
tTau> 416 pg/ml	212.493	56.439	0.446	3.765	<0.001
Apo E4	159.700	58.438	0.340	2.733	0.008
αSyn (cost.)					
Aβ 42/40 <0.062	711.559	257.650	0.404	2.762	0.009
pTau>61pg/ml	685.719	259.355	0.399	2.644	0.012
Aβ 42/Ng (cost.)					
MMSE<24	-1.031	0.236	-0.651	-4.368	<0.001
PET AD-related	-1.290	0.417	-0.362	-3.093	0.003
Aβ 42 < 0.650pg/ml	-0.763	0.133	-0.609	-5.751	<0.001
Aβ 42/40 < 0.062	-1.079	0.127	-0.762	-8.479	<0.001
pTau>61pg/ml	-0.860	0.137	-0.680	-6.285	<0.001
tTau > 416 pg/ml	-0.926	0.132	-0.684	-7.007	<0.001
Apo E4	-0.475	0.164	-0.362	-2.905	0.005
Aβ 42/α-Syn (cost.)					
MMSE < 24	-0.016	0.002	-0.539	-2.793	0.012
PET AD-related	-0.009	0.004	-0.349	-2.381	0.022
Aβ 42 < 0.650pg/ml	-0.006	0.001	-0.605	-4.927	<0.001
Aβ 42/40 < 0.062	-0-009	0.001	-0.779	-7.657	<0.001
pTau>61pg/ml	-0.008	0.001	-0.717	-6.166	<0.001
tTau > 416 pg/ml	-0.008	0.001	-0.703	-6.413	<0.001
